# Rat Lungworm Infection in Rodents across Post-Katrina New Orleans, Louisiana, USA 

**DOI:** 10.3201/eid2412.180056

**Published:** 2018-12

**Authors:** Rosalyn C. Rael, Anna C. Peterson, Bruno Ghersi-Chavez, Claudia Riegel, Amy E. Lesen, Michael J. Blum

**Affiliations:** Tulane University, New Orleans, Louisiana, USA (R.C. Rael, A.C. Peterson, B. Ghersi-Chavez, A.E. Lesen, M.J. Blum);; University of Tennessee, Knoxville, Tennessee, USA (A.C. Peterson, B. Ghersi-Chavez, M.J. Blum);; The City of New Orleans Mosquito, Termite, and Rodent Control Board, New Orleans (C. Riegel)

**Keywords:** parasites, nematode infections, urban health, meningitis/encephalitis, helminths, ecology, rats, New Orleans, Louisiana, rodents, zoonoses, United States

## Abstract

Rat lungworm (*Angiostrongylus cantonensis*), a parasitic nematode that can cause eosinophilic meningitis in humans, was first detected in New Orleans, Louisiana, USA, in the mid-1980s and now appears to be widespread in the southeastern United States. We assessed the distribution, prevalence, and intensity of *A. cantonensis* infection in New Orleans by examining lung biopsy samples of rodents trapped at 96 sites in 9 areas in Orleans Parish and 1 area in neighboring St. Bernard Parish during May 2015 through February 2017. These areas were selected to capture contrasting levels of income, flooding, and pos-disaster landscape management after Hurricane Katrina in 2005. We detected *A. cantonensis* in all areas and in 3 of the 4 rat species trapped. Overall prevalence was ≈38% but varied by area, host species, and host species co-occurrence. Infection intensity also varied by host species. These findings suggest that socioecological analysis of heterogeneity in definitive and intermediate host infection could improve understanding of health risks across the city.

Concern is increasing about the spread of rat lungworm (*Angiostrongylus cantonensis*), especially in the southeastern United States (*1*–*5*). A parasitic nematode carried by intermediate mollusk hosts and definitive rat hosts (*6*,*7*), rat lungworm can cause eosinophilic meningitis in humans who become infected by ingesting intermediate hosts or paratenic hosts, such as freshwater shrimp and frogs (*6*,*7*). *A. cantonensis* was first reported in North America from Norway rats (*Rattus norvegicus*) trapped in New Orleans, Louisiana, USA, along the Mississippi River during April 1986 through February 1987 (*8*). Later surveys suggest the parasite has since become more widespread in Louisiana. Surveys of intermediate apple snail (*Pomacea canaliculata*) hosts, for example, detected the parasite in suburban areas of New Orleans (*3*,*9*). Infections have also since been reported in nonhuman incidental mammal hosts (*9*,*10*), and 2 cases of human eosinophilic meningitis from rat lungworm infection were diagnosed in nearby areas of Louisiana (*11*,*12*). Rat lungworm also appears to have become widespread across Florida (*9*) and has been recently detected in Oklahoma (*11*).

We trapped rodents across New Orleans to characterize the current distribution, prevalence, and intensity of *A. cantonensis* infection and to determine how these aspects vary according to organismal and ecological characteristics of definitive hosts, including species co-occurrence. This study enabled us to identify factors associated with definitive host infection, which might affect transmission risk across the city and offer further insight into the global progression, surveillance, and control of rodent-associated infectious disease.

## Methods

### Study Animals

**Figure 1:**
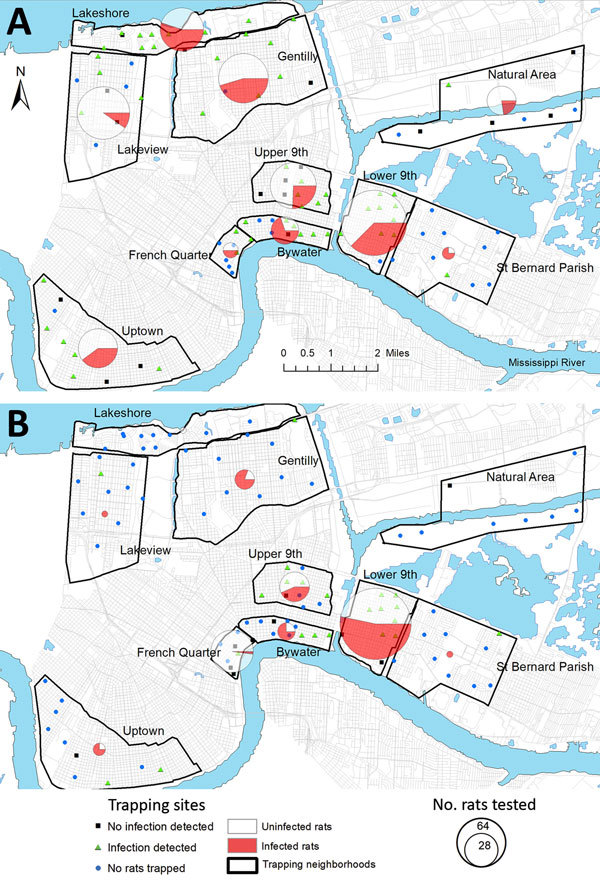
Prevalence of rat lungworm (*Angiostrongylus cantonensis*) in rodents, New Orleans, Louisiana, USA, May 2015–February 2017. A) Roof rats (*Rattus rattus*); B) Norway rats (*R. norvegicus*).

We collected rats during May 2015 through February 2017 (following Tulane University [New Orleans, LA, USA] Institutional Animal Care and Use Committee [IACUC] protocol #0451) during a quantitative population survey across 96 city blocks in 8 neighborhoods in New Orleans, a natural area in Orleans Parish, and an area in neighboring St. Bernard Parish. These areas were selected to capture contrasting levels of income, flooding, and postdisaster landscape management after Hurricane Katrina in 2005 (Figure 1; Technical Appendix Table 1) (*13*,*14*). We selected 8–10 sites in each study area by random stratification across a 1-km resolution grid spanning the city. Each site was visited 4 times during May 2015 through January 2017: once during summer and once during winter months each year, except for sites in St. Bernard Parish, which were visited only twice (summer 2016 and winter 2016–17). During each trapping period, 30 Tomahawk Live traps (Hazelhurst, WI, USA) were set in locations with potential or evident rodent activity for a minimum of 3 consecutive nights. Trapping efforts continued at each site until no additional rats were captured. Sherman traps (H.B. Sherman Traps, Inc., Tallahassee, FL, USA) were also placed at a subset of 48 sites to capture smaller rodents following the same approach (Tulane University IACUC protocol #0460).

**Table 1:** Prevalence of rat lungworm (*Angiostrongylus cantonensis*), New Orleans, Louisiana, USA, May 2015–February 2017*

Area	No. positive/no. trapped (%)				
	Roof rats	Norway rats	Cotton rats	Rice rats	Total
Uptown	16/40 (40)	3/4 (75)	–	–	19/44 (43)
Lakeview	7/72 (10)	1/1 (100)	–	–	8/73 (11)
Lakeshore	27/49 (55)	0/1 (0)	–	–	27/50 (54)
Gentilly	29/64 (45)	8/10 (80)	–	–	37/74 (50)
French Quarter	3/6 (50)	1/47 (2)	–	–	4/53 (8)
Bywater	14/20 (70)	6/8 (75)	–	–	20/28 (71)
Upper 9th	14/55 (25)	10/23 (43)	–	–	24/78 (31)
Lower 9th	42/112 (38)	71/133 (53)	0/2 (0)	–	113/247 (46)
Natural area	5/22 (23)	0/1 (0)	4/17 (24)	0/4 (0)	9/44 (20)
St. Bernard Parish	3/4 (75)	1/1 (1)	–	–	4/5 (80)
Total	160/444 (36)	101/229 (44)	4/19 (21)	0/4 (0)	265/696 (38)

*Prevalence (in parentheses) was computed for each species in each area as the total number of positive rodents divided by the number trapped. Totals represent overall prevalence and number trapped, pooled by neighborhood, species, or both (total positive/total trapped). Roof rats, *Rattus rattus*; Norway rats, *R. norvegicus*; cotton rats, *Sigmodon hispidus*; rice rats, *Oryzomys palustris*. Dashes indicate no rats were trapped.

All rodents were necropsied after euthanasia following Tulane University IACUC protocols #0451 and #0460. We recorded standard weight and length measurements, as well as species identity; sex; sexual maturity; and, in females, parity. We categorized all Norway rats and roof rats (*R. rattus*) into 3 age classes (juvenile, subadult, adult) according to body weight (*15*,*16*). Urine, lung, liver, spleen, kidney, and tail tissues were sampled and archived in −80°C freezers. We visually screened lung tissues for parasites, which were isolated, counted, and preserved in 95% ethanol. Representative lung parasites were identified through PCR (*17*) (Technical Appendix Table 2).

### Statistical Analyses

We report on the distribution and prevalence of *A. cantonensis* according to all species trapped, but additional statistical analyses considered data only from Norway rats and roof rats because of small sample sizes or because the parasite was not detected in other species. Generalized linear models (GLMs) were constructed with a quasibinomial error distribution to determine whether sex or age class was a significant predictor of infection status (i.e., infected, not infected) in Norway rats and roof rats. We ran 3 GLMs: 1 with both *Rattus* species together and 1 for each species. The same predictors were used in 3 GLMs with a quasi-Poisson distribution to examine relationships with infection intensity (i.e., number of parasites per infected rodent) in Norway rats and roof rats together and separately by species.

We used χ^2^ tests to determine whether infection prevalence in Norway rats and roof rats differed among study areas, and among sites with 1 versus >1 species present. We ran a subset of pairwise tests to compare prevalence among study areas, correcting for multiple comparisons.

We used a Kruskal-Wallis test to compare infection intensity among areas, combining data from Norway rats and roof rats. We used Mann-Whitney U tests to compare infection intensity between Norway rats and roof rats and to compare intensity among sites with 1 or >1 species present. We conducted all statistical analyses using R version 3.4.2 (https://www.r-project.org/).

## Results

### Rodent Trapping and Overall Prevalence of *A. cantonensis*

A total of 696 rats were trapped at 78 of the 96 sampling sites. Both Norway rats and roof rats were found in all 10 areas, whereas hispid cotton rats (*Sigmodon hispidus*) were found in the natural area and the Lower 9th Ward, and rice rats (*Oryzomys palustris*) were found only in the natural area.

We detected *A. cantonensis* in all 10 areas (Figure 1) and in 3 of the 4 rat species sampled. Of the 444 roof rats necropsied, 160 (36.0%) were positive for *A. cantonensis*, whereas 101 (44.1%) of 229 Norway rats, 4 (21.1%) of 19 *S. hispidus*, and 0 of 4 *O. palustris* were positive (Table 1). Prevalence differed between Norway rats and roof rats (χ^2^ = 3.810, p = 0.051). Median site-level prevalence estimates for roof rats (33.3%) and Norway rats (47.2%) (Technical Appendix Figure) were similar to those estimated by pooling rats by species and area (Table 1).

A total of 488 house mice (*Mus musculus*) were collected at 48 locations with Sherman traps. Lungworms were not detected in any house mice, affirming that they do not serve as definitive hosts (*18*).

### Geographic Variation in Prevalence

**Figure 2:**
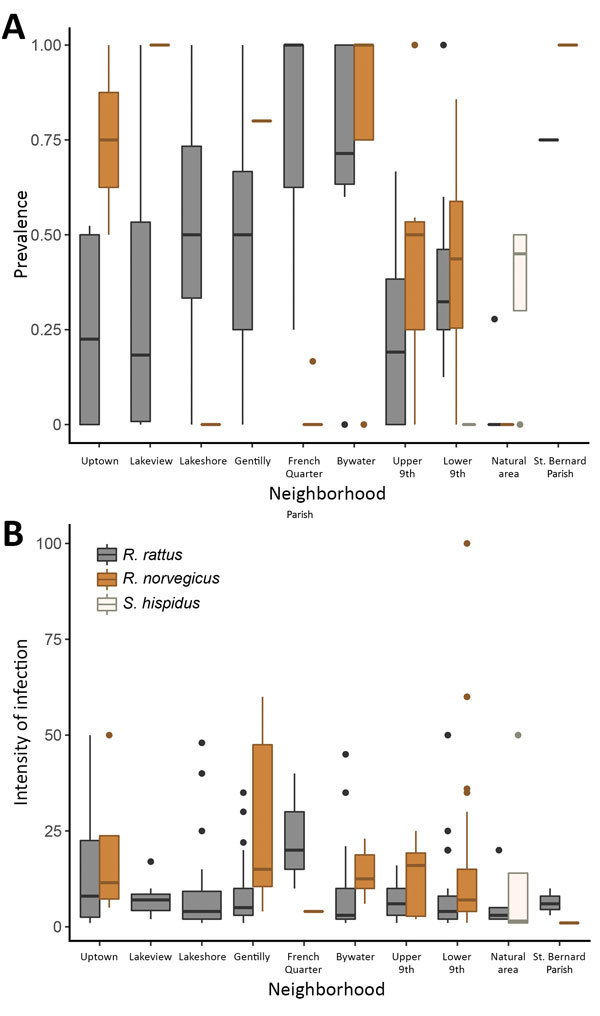
Boxplots of rat lungworm (*Angiostrongylus cantonensis*) prevalence (A) and intensity of infection (no. lungworms per infected rat) (B) showing summary statistics across sites for each area, New Orleans, Louisiana, USA, May 2015–February 2017. Tops and bottoms of boxes indicate 25th and 75th percentiles, horizontal lines within boxes indicate medians, and error bars indicate minimum and maximum values (excluding outliers). Plots were created by using the R statistical software package (https://www.r-project.org).

Excluding the St. Bernard Parish sites because of small sample sizes (n = 5), we found that overall prevalence in both Norway rats and roof rats differed among the sampled areas (χ^2^ = 81.21, p<0.001). Rats from the Bywater area exhibited the highest overall prevalence of *A. cantonensis* infection (71%), whereas rats from the French Quarter exhibited the lowest (8%) (Table 1; Technical Appendix Table 3). Most median site-level prevalence values (Figure 2, panel A) were similar to those estimated by pooling rats by species and area (Table 1).

Considering roof rats and Norway rats separately in areas with >10 samples, we found that prevalence differed among areas for both species (roof rats, χ^2^ = 46.755, p<0.0001; Norway rats, χ^2^ = 43.62, p<0.0001). Prevalence in roof rats was lowest for rats trapped in the Lakeview area (10%) and highest for those from the Bywater area (70%), although pairwise comparisons showed that prevalence differed only among a subset of the study areas (Technical Appendix Table 4). Prevalence in Norway rats was lowest for rats trapped in the French Quarter (2%) and highest among those from the Gentilly area (80%). As with roof rats, prevalence in Norway rats differed only among a subset of the study areas (Technical Appendix Table 5).

Infection prevalence was significantly lower in sites where only 1 rat species was trapped (30%, n = 267, 48 sites) than in sites that harbored multiple rat species (44%, n = 429, 30 sites) (χ^2^ = 11.654, p<0.001). However, sites with 1 species had significantly fewer rodents (mean 5.6) than sites with multiple species (mean 14.3) (Mann-Whitney U test, p<0.0001).

### Likelihood of Infection

Considering Norway rats and roof rats together, the likelihood of infection did not differ by sex (df = 656, coefficient = 0.13133, p = 0.42), but adults were significantly more likely to be infected than juveniles (df = 656, coefficient = −1.26201, p<0.0001) and subadults (df = 656, coefficient = −0.42601, p<0.05) (Technical Appendix Table 6).

When we considered species separately, we also detected differences in the likelihood of infection according to age class. The likelihood of infection in Norway rats did not differ by sex (df = 224, coefficient = −0.04621, p = 0.86), but adults were more likely to be infected than either juveniles (df = 224, coefficient = −1.88906, p = 0.003) or subadults (df = 224, coefficient = −0.82707, p = 0.007) (Technical Appendix Table 6). Among roof rats, likelihood of infection did not differ by sex (df = 427, coefficient = 0.2305, p = 0.26) or between adults and subadults (df = 427, coefficient = −0.1541, p = 0.54), but adults were more likely than juveniles to be infected (df = 427, coefficient = −0.9550, p = 0.004) (Technical Appendix Table 6).

### Infection Intensity

Infection intensity (Table 2) significantly differed between roof rats and Norway rats (Mann-Whitney U test, p<0.01). Considering Norway rats and roof rats together across all areas, infection intensity did not differ by sex (df = 240, coefficient = 0.01331, p = 0.972). Subadults had lower infection intensity than adults (df = 240, coefficient = −0.44999, p = 0.0124), but juveniles did not (df = 240, coefficient = 0.0595, p = 0.82) (Technical Appendix Table 7). Excluding the St. Bernard and French Quarter areas, each of which had only 4 infected rodents, we did not detect significant differences in infection intensity across the sampled areas (Kruskal-Wallis test, p = 0.484). Also, we found no differences in infection intensity between sites with 1 species (8.75, n = 79) versus multiple species present (11.74, n = 186) (Mann-Whitney U test, p = 0.072). Some median site-level intensities within areas (Figure 2, panel B) differed from intensities estimated by pooling all rats of each species within each area (Table 2). When we considered species separately, infection intensity did not differ according to sex or age class for Norway rats (all p>0.05) or roof rats (all p>0.05) (Technical Appendix Table 7).

**Table 2:** Intensity of infection by rat lungworm (*Angiostrongylus cantonensis*), New Orleans, Louisiana, USA, May 2015–February 2017*

Area	Intensity (no. positive)				
	Roof rats	Norway rats	Cotton rats	Rice rats	Total
Uptown	13.7 (19)	19.5 (4)	–	–	14.7 (23)
Lakeview	8.0 (7)	–	–	–	8.0 (7)
Lakeshore	8.7 (23)	–	–	–	8.7 (23)
Gentilly	8.5 (29)	27.9 (7)	–	–	12.3 (36)
French Quarter	23.3 (3)	4.0 (1)	–	–	18.5 (4)
Bywater	11.1 (12)	12.2 (5)	–	–	11.4 (17)
Upper 9th	7.1 (15)	12.3 (7)	–	–	8.7 (22)
Lower 9th	7.7 (42)	13.3 (65)	–	–	11.1 (107)
Natural area	6.4 (5)	–	13.5 (4)	–	9.6 (9)
St. Bernard Parish	6.3 (3)	1 (1)	–	–	5.0 (4)
Total	9.2 (158)	14.3 (90)	13.5 (4)	–	11.1 (252)

*Intensity was computed for each species and each area as the sum of all lungworms counted, divided by the total number of lungworm-positive rodents for which lungworms were counted (in parentheses). Totals represent overall intensity and numbers positive*_,_* pooled by neighborhood, by species, or both (total lungworms/total infected rats). Roof rats, *Rattus rattus*; Norway rats, *R. norvegicus*; cotton rats, *Sigmodon hispidus*; rice rats, *Oryzomys palustris*. Dashes indicate no rats were positive, except in the Lakeview area, where no count data were available for the 1 positive rat trapped.

## Discussion

We assessed the current distribution and prevalence of *A. cantonensis* in definitive rat hosts across New Orleans, where the parasite has been present since at least 1986 (*8*). Our overall estimate of 38% infection prevalence in New Orleans is comparable to count-based estimates reported for other areas where *A. cantonensis* is considered endemic (*19**,**20*, but see *21*). We also found *A. cantonensis* in rats across New Orleans and in neighboring St. Bernard Parish, which contrasts with the patchy distributions exhibited by other rodent-associated pathogens in cities (*22*–*25*). Although rat lungworm is present across New Orleans, infection prevalence varied according to geography and rodent host species, suggesting the risk for transmission to humans might be mediated in part by geographically variable landscape features that affect commensal rats (*13*,*14*). It is also likely, however, that the distributions of intermediate hosts and human population densities moderate transmission risk.

The first record of *A. cantonensis* in New Orleans reported lower prevalence in rat hosts than those observed in our study. An overall count-based prevalence of 18% was found for Norway rats and roof rats trapped in 1986–1987 (*8*). However, evidence of infection was found only in Norway rats; *A. cantonensis* was detected in 20 (21%) of 94 trapped Norway rats and in 0 of 19 trapped roof rats (*8*). In comparison, we found overall prevalence >18% in 8 of the 10 trapping areas in our study (Table 1), and we detected infected roof rats in all trapping areas. However, it is unclear whether the distribution of rodent host infections has changed, because the 1986–1987 surveys were limited to trapping on wharves along the Mississippi River (*8*). Thus, although we can affirm that infection prevalence varies by geography and definitive host (*8*), we cannot conclude that *A. cantonensis* became more broadly distributed across the city during the past 3 decades.

Evidence of greater prevalence and infection intensity of *A. cantonensis* in adult rats most likely reflects the increasing probability over time that an individual rat will consume an infected intermediate host and that infection becomes more evident in lung tissue. Waugh et al. (*19*) similarly reported differences in intensity and prevalence in rats from Jamaica according to size but not sex, although an earlier study found that female Norway rats were more likely to be infected (*26*). Evidence that *A. cantonensis* infection differs by host age contrasts with findings for other urban rodent–associated pathogens, including flea-vectored *Bartonella* bacteria (*25*). The finding of distinct *Bartonella* species in Norway rats and roof rats (*25*) suggests that co-occurrence does not facilitate pathogen transmission, whereas our results indicate otherwise. Contrasting patterns in the demography of definitive host infection may reflect pathogen-specific differences in transmission pathways. Further study is therefore warranted to determine the roles of definitive host abundance and diversity in pathogen transmission.

It has been more than a decade since the last diagnosed case of rat lungworm infection in the New Orleans area (*12*), which suggests that factors unrelated to rodent hosts mitigate the risk for transmission to humans. Although the most recent case in Louisiana resulted from consumption of a paratenic host (*12*), work elsewhere suggests that transmission to humans most likely occurs through accidental consumption of raw or undercooked infected snails on produce (*7*). Accordingly, the distribution and abundance of infected intermediate hosts are probably key factors affecting transmission risk, especially in cities such as New Orleans, where interest in urban agriculture is on the rise (*27*,*28*). It is also possible that risk is influenced by climate-driven spread of invasive mollusks (*29*,*30*), such as apple snails, that can serve as reservoirs (*1*,*3*). Further study of infection prevalence in intermediate hosts would thus probably improve understanding of transmission risk across New Orleans and other cities that are vulnerable to climate change.

We intentionally diagnosed infection through a visual and count-based survey to draw comparisons to historical records, but implementing complementary approaches could have provided further understanding of *A. cantonensis* infection. As has been noted in prior studies (*19*,*20*), count-based approaches probably yield conservative estimates relative to PCR-based approaches of infection. For example, in Hawaii, a count-based approach yielded a prevalence estimate of 54%, whereas PCR yielded an estimate of 100% (*20*). More extensive use of PCR-based approaches in our study probably would have afforded additional perspective on parasitism and could also have excluded possible errors due to misidentification (*31*–*33*).

Our results indicate that cross-disciplinary analysis of *A. cantonensis* infection could shed further light on the risk for transmission to humans (*34*). As has been found with other zoonoses, human risk might correspond to socioecological disparities in habitat and resources favored by infected definitive and intermediate hosts (*22*,*23*,*35*–*38*). For example, Rael et al. (*13*) detected a positive correlation between land abandonment and rat abundance in New Orleans across only low-income neighborhoods. The contrasting landscapes (*14*) and rodent assemblages (Table 1) found in the Lower 9th Ward in New Orleans and in adjacent St. Bernard Parish highlight the possibility that public health risks have been shaped by differences in postdisaster (i.e., Hurricane Katrina) land management policies (*29*). Although too few rats were trapped in St. Bernard Parish (n = 5) to confidently estimate rat lungworm prevalence (Table 1), the observed differences in rat abundance suggest that transmission risk sharply differs between the Lower 9th Ward and St. Bernard Parish. Thus, further study of associations between definitive and intermediate host infection and socioecological factors probably would better define transmission risk across the city (*14*,*39*).

Although rat lungworm is just 1 of many pathogens harbored by urban rats (*40*,*41*), citywide estimates of host infection like those presented here can provide an epidemiologic baseline that can improve understanding of infectious disease dynamics in cities. Baselines are particularly informative for cities where zoonotic pathogens are likely to (re)emerge because of shifting climate conditions (*30*,*40*,*42*) or extreme events, such as hurricanes, that can foster disease outbreaks (*13*). New Orleans unenviably straddles both sets of circumstances (*43*–*45*). Accordingly, further study of rat lungworm could help inform public health policies, surveillance programs, and intervention to safeguard the well-being of vulnerable communities in New Orleans and elsewhere.

Technical Appendix Additional methods and results for study of rat lungworm infection in rodents, New Orleans, Louisiana, USA, 2015–2017.
