# Supplementary material for: Rat Lungworm Infection in Rodents across Post-Katrina New Orleans, Louisiana, USA
Source: Emerg Infect Dis. 2018 Dec;24(12):2176–83. doi: 10.3201/eid2412.180056 (PMC6256379; doi:10.3201/eid2412.180056)
Supplement: Technical Appendix — Additional methods and results for study of rat lungworm infection in rodents, New Orleans, Louisiana, USA, 2015–2017. [file 18-0056-Techapp-s1.pdf]

Article DOI: https://doi.org/10.3201/eid2412.180056

Rat Lungworm Infection in Rodents Across Post-Katrina New Orleans, Louisiana, USA

Technical Appendix

Methods: PCR-based Species Diagnosis

Genomic DNA was extracted from a representative set of lung parasites (Technical Appendix Table 2) using the QIAGEN Blood and Tissue kit (QIAGEN, Hilden, Germany). Due to low DNA yields, genomic DNA was concentrated before PCR by spinning extractions down in a Thermo Scientific SpeedVac (Thermo Scientific, Waltham, MA, USA) to 25% of original volumes. Concentrated extractions were used to amplify a region of the cytochrome c subunit oxidase I gene (*1*). After confirming amplification through agarose gel electrophoresis, PCR products were cleaned with ExoSAP-IT (Affymetrix, Santa Clara, CA, USA) for use in sequencing reactions with 3.75 μL PCR- grade H_2O, 3.75 μL 5 μM $MgCl_2$, 1.0 μL 10 μM primer and 0.5 μL BigDye terminator (Applied Biosystems, Foster City, CA, USA). Reactions were cleaned using Sephadex columns (GE Healthcare, Chicago, IL, USA) before electrophoresis on an ABI 3730xl (Applied Biosystems, Foster City, CA, USA). Resulting sequences were trimmed for alignment and comparison to sequences archived on GenBank using a BLAST search (https://blast.ncbi.nlm.nih.gov/Blast.cgi). All sequences (GenBank accession nos. MH069730–MH069736) matched archived *A. cantonensis* sequences with 98%–99% identity.

Results: Geographic Variation in Infection Prevalence

The Bywater area exhibited the highest overall prevalence of *A. cantonensis* infection (71%), which was significantly different from prevalence in the French Quarter (8%, $\chi^2 = 32.86$, $p<0.0001$), the natural area (20%, $\chi^2 = 16.43$, $p<0.0001$), Lakeview (11%, $\chi^2 = 33.98$, $p<0.0001$), and the Upper 9th Ward (31%, $\chi^2 = 12.41$, $p = 0.0004$) (all other comparisons, $p>0.00139$) (Technical Appendix Table 3). The French Quarter exhibited the lowest overall prevalence of infection (8%), which significantly differed from the Uptown (43%, $\chi^2 = 14.96$, p

= 0.0001), Lower 9th Ward (46%, χ^2 = 25.19, p<0.0001), Gentilly (50%, χ^2 = 23.55, p<0.0001), Lakeshore (54%, χ^2 = 24.23, p<0.0001), and Bywater (71%, χ^2 = 32.86, p<0.0001) areas (all other comparisons, p>0.00139) (Technical Appendix Table 3).

Prevalence in *R. rattus* trapped in the Lakeview area (10%) significantly differed from prevalence in *R. rattus* trapped in the Lower 9th Ward (38%, χ^2 = 15.92, p<0.0001), Uptown (40%, χ^2 = 12.65, p = 0.0004), Gentilly (45%, χ^2 = 20.26, p<0.0001), and Bywater (70%, χ^2 = 28.95, p<0.0001) areas (Technical Appendix Table 4). Prevalence in *R. rattus* also differed between the Bywater (70%) and Upper 9_{th} Ward (25%, χ^2 = 10.61, p = 0.001) areas (all other comparisons p>0.00179) (Technical Appendix Table 4).

Prevalence in *R. norvegicus* trapped in the French Quarter (2%) significantly differed from prevalence in the Upper 9th Ward (43%, χ^2 = 16.94, p<0.0001), Lower 9th Ward (53%, χ^2 = 35.91, p<0.0001), and Gentilly (80%, χ^2 = 31.977, p<0.0001) areas (all other comparisons p>0.00833) (Technical Appendix Table 5).

Reference

1. Eamsobhana P, Lim PE, Solano G, Zhang H, Gan X, Yong HS. Molecular differentiation of *Angiostrongylus* taxa (Nematoda: Angiostrongylidae) by cytochrome c oxidase subunit I (COI) gene sequences. Acta Trop. 2010;116:152–6. PubMed http://dx.doi.org/10.1016/j.actatropica.2010.07.005

Technical Appendix Table 1. Income level, Hurricane Katrina flooding, and rat species richness across trapping areas, New Orleans, Louisiana, USA, 2015–2017

			No. sites‡	
Area	Income*	Flooding†	With 1 sp.	With >1 sp.
Uptown	High	No	5	4
Lakeview	High	Yes	5	1
Lakeshore	High	No	10	0
Gentilly	Low	Yes	8	1
French Quarter	High	No	4	2
Bywater	Low	No	5	3
Upper 9th	Low	Yes	4	6
Lower 9th	Low	Yes	1	9
Natural area	NA	Yes	2	5
St. Bernard	High	Yes	2	0

*Median yearly household income threshold for "high" vs. "low" designation is $42,196 (2007–2011 average, US Census).
†Flooding categorization (yes/no) indicates whether the area sustained >2-ft. flooding from Hurricane Katrina (2005).
‡Number of sites within each area where rat species were trapped.

Technical Appendix Table 2. Species, area, and season trapped for rats from which lung parasites were sampled for PCR-based species identification, New Orleans, Louisiana, USA, 2015–2017*

Species	Area	Season trapped
N	Lower 9th	Summer 2015
N	Upper 9th	Summer 2016
R	Lower 9th	Summer 2015
R	Lower 9th	Winter 2015–16
R	Upper 9th	Summer 2015
R	Upper 9th	Summer 2016
R	Gentilly	Summer 2016

*N, Norway rat (*Rattus norvegicus*); R, roof rat (*R. rattus*). One lungworm was sampled from each rat.

Technical Appendix Table 3. Post hoc χ^2 overall neighborhood prevalence comparisons for *Rattus rattus* and *R. norvegicus* combined, New Orleans, Louisiana, USA, 2015–2017

Comparison	p value
Uptown vs. Lakeview	0.0002*
Uptown vs. Lakeshore	0.4008
Uptown vs. Gentilly	0.5985
Uptown vs. French Quarter	0.0001*
Uptown vs. Bywater	0.0355
Uptown vs. Upper 9th	0.2377
Uptown vs. Lower 9th	0.8801
Uptown vs. natural area	0.0394
Lakeview vs. Lakeshore	<0.0001*
Lakeview vs. Gentilly	<0.0001*
Lakeview vs. French Quarter	0.7364
Lakeview vs. Bywater	<0.0001*
Lakeview vs. Upper 9th	0.0055
Lakeview vs. Lower 9th	<0.0001*
Lakeview vs. natural area	0.2539
Lakeshore vs. Gentilly	0.7994
Lakeshore vs. French Quarter	<0.0001*
Lakeshore vs. Bywater	0.2049
Lakeshore vs. Upper 9th	0.0149
Lakeshore vs. Lower 9th	0.3625
Lakeshore vs. natural area	0.0018
Gentilly vs. French Quarter	<0.0001*
Gentilly vs. Bywater	0.0851
Gentilly vs. Upper 9th	0.0243
Gentilly vs. Lower 9th	0.6100
Gentilly vs. natural area	0.0028
French Quarter vs. Bywater	<0.0001*
French Quarter vs. Upper 9th	0.0030
French Quarter vs. Lower 9th	<0.0001*
French Quarter vs. natural area	0.1191
Bywater vs. Upper 9th	0.0004*
Bywater vs. Lower 9th	0.0174
Bywater vs. natural area	<0.0001*
Upper 9th vs. Lower 9th	0.0275
Upper 9th vs. natural area	0.3080
Lower 9th vs. natural area	0.0030

*With Bonferroni corrections, differences are significant at the 95% level for p<0.00139.

Technical Appendix Table 4. Pos -hoc χ^2 overall neighborhood prevalence comparisons for *Rattus rattus* only, New Orleans, Louisiana, USA, 2015–2017

Comparison	p value
Uptown vs. Lakeview	0.0004*
Uptown vs. Lakeshore	0.2282
Uptown vs. Gentilly	0.7425
Uptown vs. Bywater	0.0552
Uptown vs. Upper 9th	0.1997
Uptown vs. Lower 9th	0.9284
Uptown vs. natural area	0.2737
Lakeview vs. Lakeshore	<0.0001*
Lakeview vs. Gentilly	<0.0001*
Lakeview vs. Bywater	<0.0001*

Comparison	p value
Lakeview vs. Upper 9th	0.0337
Lakeview vs. Lower 9th	0.0001*
Lakeview vs. natural area	0.2169
Lakeshore vs. Gentilly	0.4000
Lakeshore vs. Bywater	0.3826
Lakeshore vs. Upper 9th	0.0039
Lakeshore vs. Lower 9th	0.0570
Lakeshore vs. natural area	0.0228
Gentilly vs. Bywater	0.0946
Gentilly vs. Upper 9th	0.0397
Gentilly vs. Lower 9th	0.3917
Gentilly vs. natural area	0.1060
Bywater vs. Upper 9th	0.0011*
Bywater vs. Lower 9th	0.0138
Bywater vs. natural area	0.0057
Upper 9th vs. Lower 9th	0.1691
Upper 9th vs. natural area	1.0000
Lower 9th vs. natural area	0.2788

*With Bonferroni corrections, differences are significant at the 95% level for p<0.00179.

Technical Appendix Table 5. Post hoc χ^2 comparisons of overall neighborhood prevalence comparisons for *Rattus norvegicus*, New Orleans, Louisiana, USA, 2015–2017

Comparison	p value
Gentilly vs. French Quarter	<0.0001*
Gentilly vs. Upper 9th	0.1197
Gentilly vs. Lower 9th	0.1927
French Quarter vs. Upper 9th	<0.0001*
French Quarter vs. Lower 9th	<0.0001*
Upper 9th vs. Lower 9th	0.5145

*With Bonferroni corrections, differences are significant at the 95% level for p<0.00833.

Technical Appendix Table 6. Infection prevalence by sex and age class, New Orleans, Louisiana, USA, 2015–2017*

	Sex, prevalence, % (total no. rats)		Age class, prevalence, % (total no. rats)		
Species	M	F	Juvenile	Subadult	Adult
N	43 (112)	45 (113)	15 (20)	34 (73)	54 (134)
R	34 (231)	39 (199)	22 (85)	39 (257)	44 (91)
N & R	37 (343)	41 (312)	21 (105)	38 (330)	50 (225)

Prevalence was computed as the total number of lungworm-positive rodents for each species in each sex or age class, divided by the number trapped for each category. N, Norway rat (*Rattus norvegicus*); R, roof rat (*R. rattus*). Prevalence estimates for both species combined were computed by pooling the total number of lungworm-positive individuals in each category, and dividing by the total number trapped for that category.

Technical Appendix Table 7. Intensity of infection by rodent sex and age class, New Orleans, Louisiana, USA, 2015–2017*

	Sex, intensity (no. positive rats)		Age class, intensity (no. positive rats)		
Species	M	F	Juvenile	Subadult	Adult
N	13.98 (46)	15.22 (45)	4.67 (3)	9.14 (22)	16.76 (67)
R	8.59 (74)	9.33 (75)	14.35 (17)	7.59 (96)	10.33 (39)
N & R	10.66 (120)	11.54 (120)	12.90 (20)	7.88 (118)	14.40 (106)

*Intensity was computed as the sum of all lungworms counted for each species in each age class or sex, divided by the total number of lungworm-positive rats for which lungworms were counted for each category. Intensities for both species combined were computed by pooling the total lungworm count for Norway and roof rats in each category, and dividing by the total number of lungworm-positive rats. N, Norway rat (*Rattus norvegicus*); R, roof rat (*R. rattus*).

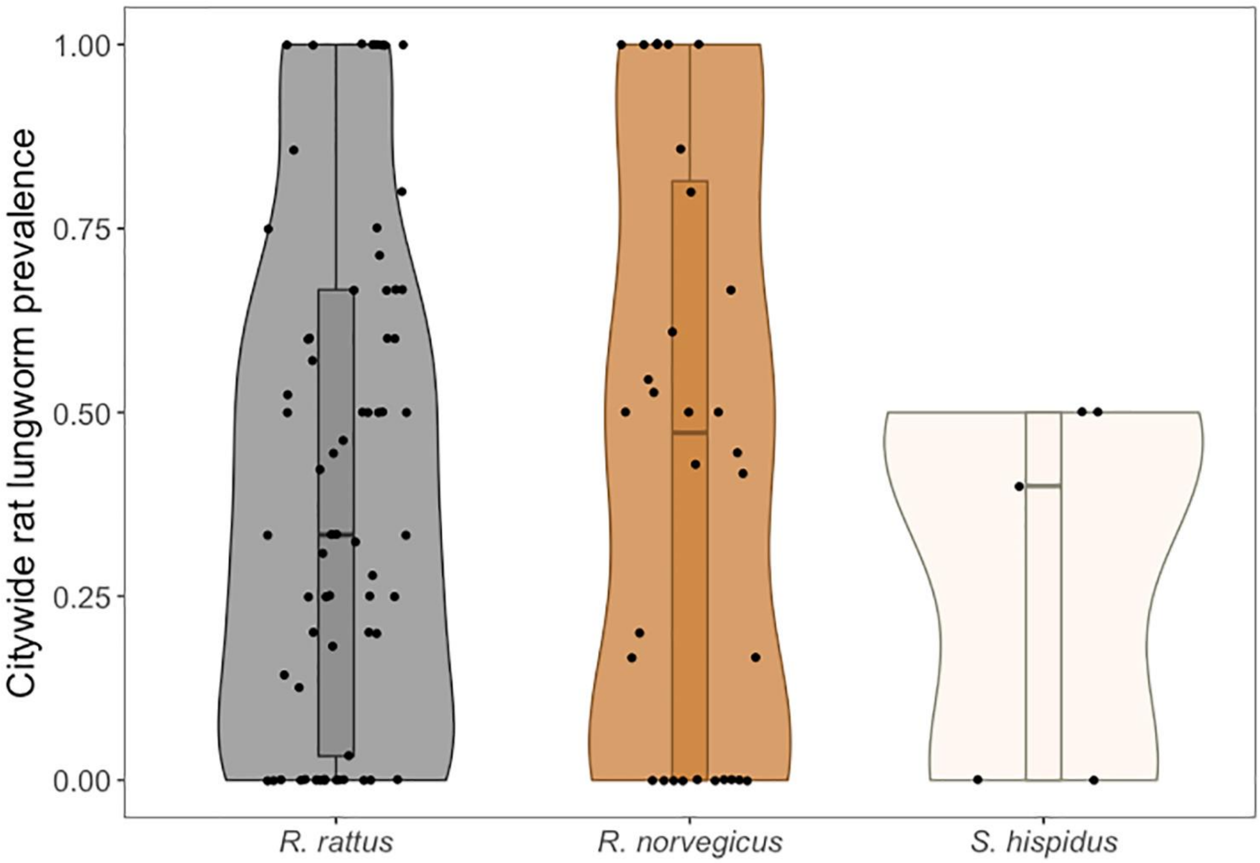

Technical Appendix Figure. Site-level prevalence for each species, New Orleans, Louisiana, USA, 2015–2017. The distribution of prevalence across sites (dots) according to definitive host species illustrates that prevalence varied widely across sites within species. This is in part due to differences in the number of individuals trapped per species at each site.
